# IL‐17A Induces Circadian Disruptions Through the Epigenetic Repression of BMAL1 in Mice With Alzheimer's Disease

**DOI:** 10.1111/jcmm.70546

**Published:** 2025-04-10

**Authors:** Ting Liu, Tian Mao, Jinxuan Fan, Yanjun Shen, Lingxia Xue, Kaili Du, Yang Li, Li Wang, Xiaohui Wang

**Affiliations:** ^1^ Department of Pathology School of Basic Medical Science, Shanxi Medical University Taiyuan Shanxi People's Republic of China; ^2^ Key Laboratory of Cellular Physiology Ministry of Education, Shanxi Medical University Taiyuan Shanxi People's Republic of China

**Keywords:** Alzheimer's disease, BMAL1, circadian rhythm, DNA methylation, IL‐17A

## Abstract

Circadian disruptions and neuroinflammation impact nearly all people with Alzheimer's disease (AD), but their relationships with each other and the impact of their interaction on AD remain to be addressed. Here, we found that amyloid (A)‐β treatment downregulated brain and muscle aryl hydrocarbon receptor nuclear translocator‐like (BMAL) 1 through the hypermethylation of its promoter region in HT22 cells and that the inhibition of DNA methylation ameliorated circadian rhythm disorders and restored BMAL1 protein expression by reversing its hypermethylation in APPswe/PSEN1dE9 (APP/PS1) mice. Critically, increased levels of interleukin (IL)‐17A contributed to BMAL1 downregulation through the hypermethylation of its promoter region, thus leading to circadian disruptions in APP/PS1 mice. Moreover, we revealed that the mitogen‐activated protein kinase (MAPK) pathway was responsible for IL‐17A‐induced DNA methyltransferase (DNMT) 1 upregulation. Taken together, we elucidate a new mechanism connecting IL‐17A with altered DNA methylation of *Bmal1*, which results in circadian disturbances in an AD mouse model.

**Abbreviations**
5‐Aza: 5‐azacytidine
5mC: 5‐methylcytosine
AD: Alzheimer's disease
APP/PS1: APPswe/PSEN1dE9
Aβ: amyloid‐β
BMAL1: brain and muscle aryl hydrocarbon receptor nuclear translocator‐like 1
BSP: bisulphite genomic sequencing PCR
CLOCK: circadian locomotor output cycles kaput
CRY1: cryptochromes circadian regulator 1
CT: circadian time
DD: dark–dark
DNMT1: DNA methyltransferase 1
icv: intracerebroventricular
IL‐17A: interleukin‐17A
JAK: Janus kinase
LD: light–dark
mAb: monoclonal antibody
MAPK: mitogen‐activated protein kinase
MSP: methylation specific PCR
PER2: period circadian regulator 2
STAT: signal transducer and activator of transcription
TETs: ten‐eleven translocation methylcytosine dioxygenases

## 1 Introduction

As the seventh‐leading cause of death in humans, Alzheimer's disease (AD) is a devastating neurodegenerative disorder with high morbidity and mortality rates [1]. In addition to the aggregation of amyloid (A)‐β and hyperphosphorylated tau protein in the brain, circadian rhythm disruptions are a common symptom experienced by patients with AD [2]. An increasing body of research indicates a bidirectional relationship between circadian homeostasis and the progression of neurodegenerative disorders [3, 4]. Moreover, emerging evidence suggests that circadian rhythm disturbances occur in the preclinical stages of AD and may exacerbate its pathology [5, 6, 7]. Circadian disruptions have been reported to increase the accumulation of Aβ. Moreover, Aβ accumulation and the resulting neuronal and synaptic injury could drive circadian dysfunction during the preclinical phase of AD, thus triggering a vicious cycle between AD progression and circadian disruptions [6]. However, the underlying mechanisms that mediate circadian dysfunction in AD are not well defined.

Brain and muscle aryl hydrocarbon receptor nuclear translocator‐like (BMAL) 1 is a core positive component of this circadian system and acts as a transcriptional activator in the auxiliary loop [8]. Experimental studies in mice suggest that Aβ accumulation could contribute to circadian disruption by downregulating BMAL1 [9, 10]. Interestingly, our previous research revealed that the *Bmal1* mRNA level was significantly decreased in Aβ_31‐35_‐injected mice, thus leading to circadian rhythm disorders [11, 12]. Therefore, it is imperative to identify the specific molecular mechanism of Aβ‐induced *Bmal1* mRNA downregulation.

Alterations in epigenetic mechanisms could also contribute to AD progression via the dysregulation of the circadian system [8]. The rhythmic methylation of *Bmal1* is altered in human brain samples and fibroblasts from AD patients and is correlated with cognitive impairment and circadian disturbances [13, 14]. Furthermore, 5‐azacytidine (5‐Aza) can induce *Bmal1* transcription through DNA demethylation in the *Bmal1* promoter and stimulate the transcription of *period circadian regulator* (*Per*)*2* and *cryptochrome circadian regulator* (*Cry*)*1*, indicating that DNA methylation of the *Bmal1* gene is critical for circadian disruptions [15]. DNA methylation is usually mediated by DNA methyltransferases (DNMTs), which are categorised into DNMT1, DNMT2 and DNMT3 [16]. Research has shown that the expression of DNMT1 is increased in a model of high‐methionine diet‐induced AD‐like symptoms and that *Dendrobium nobile* Lindl. Alkaloids (DNLA), the active ingredient originally extracted from the traditional Chinese herbal medicine *Dendrobium nobile*, can alleviate learning and memory dysfunction by DNMT1 downregulation [17]. Nevertheless, further research is needed to clarify the specific mechanisms of DNMT1 upregulation in AD.

It has been shown that IL‐6 promotes elevated DNMT1 protein expression in the hippocampal tissue of APP/PS1 mice [18]. The proinflammatory factor IL‐17A is known to induce IL‐6 secretion [19, 20], and IL‐17A plays an important role in AD‐related neuroinflammation [21, 22]. Numerous studies have demonstrated that the level of interleukin (IL)‐17A is significantly increased in AD patients [23] and that the neutralisation of IL‐17A prevents cognitive impairments and synaptic dysfunction in the early stages of ad [24]. IL‐17A promotes the progression of AD by increasing neuroinflammation and accelerating the deposition of Aβ_1‐42_ in AD model mice [25]. Moreover, IL‐17A induces neuroinflammation through its interaction with the specific receptor IL‐17R, which is highly expressed in the CA1 region of the hippocampus [26, 27]. Thus, neuroinflammation is increasingly recognised as a major risk factor for the pathogenesis of AD, but its relationship with the circadian rhythm is not yet well defined. In this study, we investigated whether increased IL‐17A levels in APPswe/PSEN1dE9 (APP/PS1) mice could induce circadian rhythm disruption and its specific epigenetic mechanisms both in vivo and in vitro, and the results will serve as the basis for potential AD treatment strategies.

## 2 Materials and Methods

### 2.1 Animals

APP/PS1 and C57BL/6 (WT) mice (18–22 g) were purchased from the Model Animal Research Center of Nanjing University, PR China. Mice were maintained under a 12‐h light/dark cycle with food and water available until 6 months. All study procedures were performed in strict accordance with the recommendations in the Guide for the Care and Use of Laboratory Animals of the National Institutes of Health and were approved by the Experimental Animal Ethics Committee of Shanxi Medical University (SYXK2015‐0001).

The circadian rhythmicity, *Bmal1* and *Dnmt1* mRNA and protein levels were examined in WT and APP/PS1 mice. The mice were randomly divided into two groups as follows: WT group (*n* = 6) and APP/PS1 group (*n* = 6). The effects of 5‐Aza on circadian rhythmicity, *Bmal1* methylation levels, *Dnmt1* and *Bmal1* transcript and expression levels were observed in WT and APP/PS1 mice. The groups were as follows: WT group (*n* = 6), APP/PS1 group (*n* = 6), APP/PS1 + DMSO group (*n* = 6) and APP/PS1 + 5‐Aza group (*n* = 6). The effects of IL‐17A neutralising monoclonal antibody (mAb) on circadian rhythmicity, *Dnmt1* and *Bmal1* transcript and expression levels and *Bmal1* methylation levels were determined in WT and APP/PS1 mice. The mice were randomly assigned to 4 groups: WT group (*n* = 6), APP/PS1 group (*n* = 6), APP/PS1 + IgG isotype group (*n* = 6) and APP/PS1 + IL‐17A Ab group (*n* = 6).

### 2.2 In Vivo Drug Administration and Adeno‐Associated Virus Injection

Six‐month‐old APP/PS1 mice were anaesthetised by inhalation of 1.5% isoflurane (RWD Life Science, Shenzhen, China) in 100% oxygen and then placed on a stereotaxic frame. For prolonged administration, 5‐Aza (S1782, Selleck, Houston, USA) or IL‐17A neutralising mAb (BE0173, BioXCell, New Hampshire, USA) was intracerebroventricularly (icv) injected into the left lateral ventricle of mice using osmotic micropumps before they were placed on a wheel‐running device. The ALZET Brain Infusion Kit used for prolonged administration was established as described in previous studies [24, 28]. The osmotic pumps were filled with recombinant mouse 5‐Aza (676.2 ng/animal, for 3 weeks at a constant rate of 0.11 μL/h)/IL‐17A mAb (682.5 μg/animal, for 3 weeks at a constant rate of 0.11 μL/h) or DMSO/IgG isotype control and were implanted subcutaneously along the back of the neck. An infusion cannula (0008851, ALZET Osmotic Pumps, Cupertino, California 95014, USA) connected to the micropump was placed in the left lateral ventricle at AP −1.0, ML +0.5 and DV −3.0 relative to the bregma. The doses of 5‐Aza [29, 30] and IL‐17A mAb [24] were determined based on previous studies. These mice received 32.2 ng/day of 5‐Aza or 32.50 μg/day of IL‐17A mAb for 21 days (Figures 4A and 7A).

The BMAL1 overexpressing virus (AAV‐CMV‐BMAL1‐mCherry) and control virus (AAV‐CMV‐mCherry) were obtained from Hanbio Biotechnology Co. Ltd. (Shanghai, China). Three weeks before the wheel‐running behavioural test, AAV vectors were stereotaxically injected into the bilateral hippocampus (AP −2.0, ML ±1.8 and DV −1.8) at a rate of 0.2 μL/min. After the injection, the microinjector was held in place for 10 min before being withdrawn.

### 2.3 Wheel‐Running Behavioural Test

A wheel‐running behavioural test was employed to examine the effects of 5‐Aza/IL‐17A neutralising mAb or BMAL1 overexpression on the endogenous circadian rhythm of the mice. Each group of 6‐month‐old mice was placed on a wheel‐running device. The light setting of the cages was 12 h of light: 12 h of dark (Light–Dark, LD) for 1 week to develop a regular sleep‐exercise pattern and then transformed into constant darkness (Dark–Dark, DD) for 2 weeks. The time during the DD conditions was defined as the circadian time (CT), and CT12 designated the beginning of activity. Upon the termination of wheel running, the mice were decapitated at CT12, and the hippocampus was isolated immediately for further research. Wheel‐running activity data were collected using the Clocklab program (Actimetrics Inc. Wilmette, IL, USA) and analysed by Clocklab analysis.

### 2.4 Cell Culture and Administration

The mouse hippocampal neuronal cell line HT22 cells were cultured in Dulbecco's modified Eagle medium (C11995500BT, Gibco, New York, USA) supplemented with 10% fetal bovine serum (10270–106, Gibco, New York, USA) and 1% penicillin–streptomycin solution (P1300, Solarbio, Beijing, China). The cells were grown in a humidified incubator with 5% CO_2_ at 37°C. Cell synchronisation was performed as follows: the culture medium was replaced with starvation medium supplemented with 1% fetal bovine serum and 1% penicillin–streptomycin. After 1 h of starvation, the circadian rhythm of the HT22 cells was considered synchronised to CT0. The synchronised cells, which were cultured in serum‐free medium containing 5 μM Aβ_1‐42_ (52487, GL Biochem Ltd., Shanghai, China), 250 ng/mL recombinant mouse (rm) IL‐17A (CX14, Novoprotein, Suzhou, China) or 10 μM 5‐Aza for n hours were denoted as CTn. The cells were harvested at CT0, CT4, CT8, CT12, CT16 and CT20. The doses of rmIL‐17A [31] and 5‐Aza [30] were determined based on previous studies and our in vitro results, as shown in Figure S3. The dosage of Aβ was determined according to the cell viability results shown in Figure S1.

### 2.5 Aβ Oligomer (AβO) Preparation

Human Aβ_1‐42_ was dissolved in hexafluoro‐2‐isopropanol (HFIP; Macklin) to obtain a 1 mM solution. HFIP was subsequently eliminated using a SpeedVac (Thermo Scientific) to obtain a clear peptide film. The film could be stored at −80°C for 6 months. Before use, the Aβ film was prepared in DMSO and sonicated for 10 min to ensure thorough resuspension, followed by dilution to 100 μM with DMEM. The mixture was incubated for 24 h and then centrifuged at 13,000 rpm for 10 min at 4°C. Eventually, Aβ oligomers were obtained in the supernatant.

### 2.6 Quantitative Real‐Time PCR

Total RNA was extracted from cells or hippocampal tissue using TRIzol (TCH022, Takara, Japan), and complementary DNA (cDNA) was synthesised with a PrimeScript RT kit (RR036A, Takara, Japan) according to the manufacturer's protocol. Real‐time PCR of target mRNAs was performed using an RT‐qPCR rapid qPCR hybrid kit with TB Green (RR820A, Takara, RR430A, Japan) on a Real‐time System (Applied Biosystems). Gene expression was normalised to that of GAPDH and compared with that of the control sample to calculate relative expression values using the 2^−ΔΔCt^ method. The primers used are listed in Table 1.

**TABLE 1:** Primers used for qRT–PCR, BSP and MSP.

qRT–PCR	
Gene	Primer sequence (5′‐3′)
*Bmal1*	F: ACGACATAGGACACCTCGCAGA
	R: TCCT‐TGGTCCACGGGTTCA
*Dnmt1*	F: TCACTTGGACGAGGACGAG‐GAC
	R: TACCTGCTCTGGCTCTGCTTCC
*Gapdh*	F: AAATGGTGAAGGTCGGTGTGAAC
	R: CAACAATCTCCACTTTGCCACTG
**Bisulfite genomic sequencing PCR (BSP)**	
F: GGGTTTTAGGAAGGTTT‐TGTTATTT	
R: ACTAATTTACCTACTACTTTCCCTTCAC	
**Methylation sepecific PCR (MSP)**	
**Primer type**	**Primer sequence (5′‐3′)**
Methylated	F: GGTTGGGGTAAGAAATTTATAGAGC
	R: TAAACTAACCAATCGAATAACCGAT
Unmethylated	F: TTGGGGTAAGAAATTTATAGAGTGT
	R: TAAACTAACCAATCAAATAACCAAT

### 2.7 Western Blot Analysis

Hippocampal tissue from each mouse or cell line in six‐well plates was sonicated in radioimmunoprecipitation assay (RIPA) buffer (AR102‐100, BOSTER, Wuhan, China) containing 1% protease inhibitor (AR1178, BOSTER, Wuhan, China) and phosphate inhibitor (AR1183, BOSTER, Wuhan, China). Protein concentration was quantified with a BCA protein assay kit (AR0146, BOSTER, Wuhan, China). Samples containing equal amounts of protein (25 μg) were separated by 8%–10% sodium dodecyl sulphate polyacrylamide gel electrophoresis (SDS–PAGE) and transferred onto polyvinylidene difluoride (PVDF) membranes (IPVH00010, Millipore, Darmstadt, Germany). The membranes were blocked with 10% non‐fat milk in Tween/Tris‐buffered salt solution (TBS, 20 mM Tris–HCl, pH 7.5, 0.15 M NaCl and 0.1% Tween‐20) for 1 h and incubated with primary antibodies at 4°C overnight. Afterwards, the samples were incubated with horseradish peroxidase‐conjugated goat anti‐mouse or anti‐rabbit IgG (1:5000, ZSGB‐BIO, Beijing, China) for 1 h at room temperature. Meilunbio fg supersensitive ECL luminescence reagent (MA0186, Meilunbio, Dalian, China) was used to detect the signals, and the blots were visualised using a Bio‐Rad microimaging system (FluorChem E, Silicon Valley, USA). The signals for the bands were quantified by densitometry analysis using ImageJ Software after the blots were imaged. The primary antibodies used were as follows: BMAL1 (1:1000, D2L7G, Cell Signaling Technology, Danvers, MA), DNMT1 (1:1000, D63A6, Cell Signaling Technology, Danvers, MA), P‐AKT (Ser 473 1:1000, 9271, Cell Signaling Technology, Danvers, MA), AKT (1:1000, 2938, Cell Signaling Technology, MA), P‐MAPK (Thr 202/Tyr 204, 1:1000, 4370, Cell Signaling Technology, MA), MAPK (1:1000, 4695, Cell Signaling Technology, MA), GAPDH (bsm‐33033M, Bioss, Beijing, China), β‐actin (1:10,000, 60008‐1‐Ig, Proteintech, Rosemont, IL, USA), β‐tubulin (1:20,000, 66240‐1‐Ig, Proteintech, Rosemont, IL, USA).

### 2.8 BSP

Genomic DNA was extracted from the harvested cells, after which the CpG island methylation status was determined using bisulphite genomic sequencing of the CpG islands. After treatment with bisulphite (59824, Epitech Bisulfite Kit, Qiagen, Massachusetts, USA), modified DNA was amplified with BSP primers according to the manufacturer's instructions (R110Q, Takara, Japan). After agarose gel electrophoresis, the gel was collected for further DNA sequencing. The BSP primer sequences are listed in Table 1.

### 2.9 MSP

Genomic DNA was extracted from cells using a TIANamp Genomic DNA Kit purchased from Tiangen according to the manufacturer's instructions. The genomic DNA was incubated with sodium bisulphite for the conversion of all unmodified cytosine to uracil using the EpiTect Fast DNA Bisulfite Kit purchased from Qiagen. PCR was performed on bisulphite‐converted DNA using the primers listed in Table 1. The PCR conditions were as follows: 95°C for 3 min; 40 cycles of 98°C for 10 s, incubation at the specific annealing temperature (unmethylated, 55°C; methyl, 55°C) for 30 s and 72°C for 30 s; and a final extension of 7 min at 72°C.

The methylated region was amplified with primers specific for either methylated or modified unmethylated DNA using a special experimental kit (R110Q, Takara, Beijing, China) with specific MSP primers.

### 2.10 Cerebrospinal Fluid (CSF) Collection

CSF was collected as described in previous studies [32, 33] Briefly, upon the completion of the wheel‐running behavioural test, the mice were anaesthetised with pentobarbital sodium (50 mg/kg, intraperitoneally) and then placed on a stereotaxic instrument. A sagittal incision was made inferior to the occiput, and muscles were held apart with microretractors, allowing the exposure of the cisterna magna by blunt dissection with forceps. CSF was extracted from the cisterna magna with a capillary tube connected to a 3 mL syringe. CSF was kept in a low‐protein binding tube on ice, and the supernatant was collected after centrifugation at 1500 rpm for 10 min and then stored at −80°C until use.

### 2.11 Enzyme‐Linked Immunosorbent Assay

IL‐17A levels in CSF harvested from mice were measured using an ELISA kit (EK0431, BOSTER, Wuhan, China) according to the manufacturer's instructions. A microplate reader (Infinite F50, Tecan) was used to measure the absorbance at 450 nm for data collection and analyses.

### 2.12 Immunohistochemical Staining

Four percent paraformaldehyde (PFA)‐fixed, paraffin‐embedded brain tissue was sectioned (5 mm thick), and the sections were dehydrated and deparaffinised in xylene solution, followed by antigen retrieval with EDTA buffer. After being blocked with 10% goat serum at room temperature for 10 min, the sections were stained with primary antibody against DNMT1 (1:200) at 4°C overnight. The sections were subsequently incubated with secondary antibody at 37°C for 1 h, and the signals were developed in 3,3′‐diaminobenzidine (DAB) substrate solution, followed by haematoxylin counterstaining, 1% hydrochloric acid alcohol‐mediated differentiation and sealing with neutral resin.

### 2.13 Immunofluorescent Staining

Mice were transcardially perfused with 0.9% sodium chloride and 4% PFA. The mouse brains were isolated and fixed with 4% PFA for 24 h at 4°C. The brains were then sectioned coronally at a thickness of 20 μm using a freezing microtome (CM1950 Clinical Cryostat, Leica Biosystems, Wetzlar, Germany). The sections were then blocked with 10% goat serum at room temperature and incubated overnight at 4°C with a primary antibody against BMAL1 (DF10308, Affinity, Jiangsu, China). The next day, the sections were incubated with secondary antibodies for 2 h at room temperature. The secondary antibodies utilised were DyLight 488‐conjugated AffiniPure goat anti‐rabbit IgG (H + L) (DF10308, 1:200, BOSTER, Wuhan, China). Finally, the slices were mounted with mounting media containing DAPI (0100‐20, SouthernBiotech, Carlsbad, USA). Fluorescence images were captured on a confocal microscope (OLYMPUS FV3000) for acquisition. Images of the immunofluorescently stained samples were analysed with Image J software (NIH, Bethesda, Maryland 20810, USA). TIFF images were opened in Image J and converted to black‐and‐white 8‐bit images. The threshold of the image was adjusted to best cover the selected region of interest and then the region was measured and recorded. The mean grey value was automatically calculated using Image J.

### 2.14 Statistical Analysis

The data are represented as means ± SEMs. Immunoblots were quantified using Image J software. GraphPad Prism version 9.5.1 (GraphPad Software, La Jolla, CA, USA) was used to analyse the data. Statistical analyses were performed using one‐way or two‐way analysis of variance (ANOVA) followed by pairwise multiple comparison procedures using the Bonferroni test. The LSD‐*t* test was used for comparisons between groups. JTK_CYCLE [34] with a period set to 24 was employed to detect rhythmic changes in *Bmal1* mRNA and protein levels. This algorithm has been widely used to test rhythms in humans and animals [35, 36]. A nonparametric rank correlation was used to detect significant rhythms. *p* < 0.05 was considered statistically significant.

## 3 Results

### 3.1 Circadian Rhythm Disturbances Are Observed in APP/PS1 Mice

**FIGURE 1:**
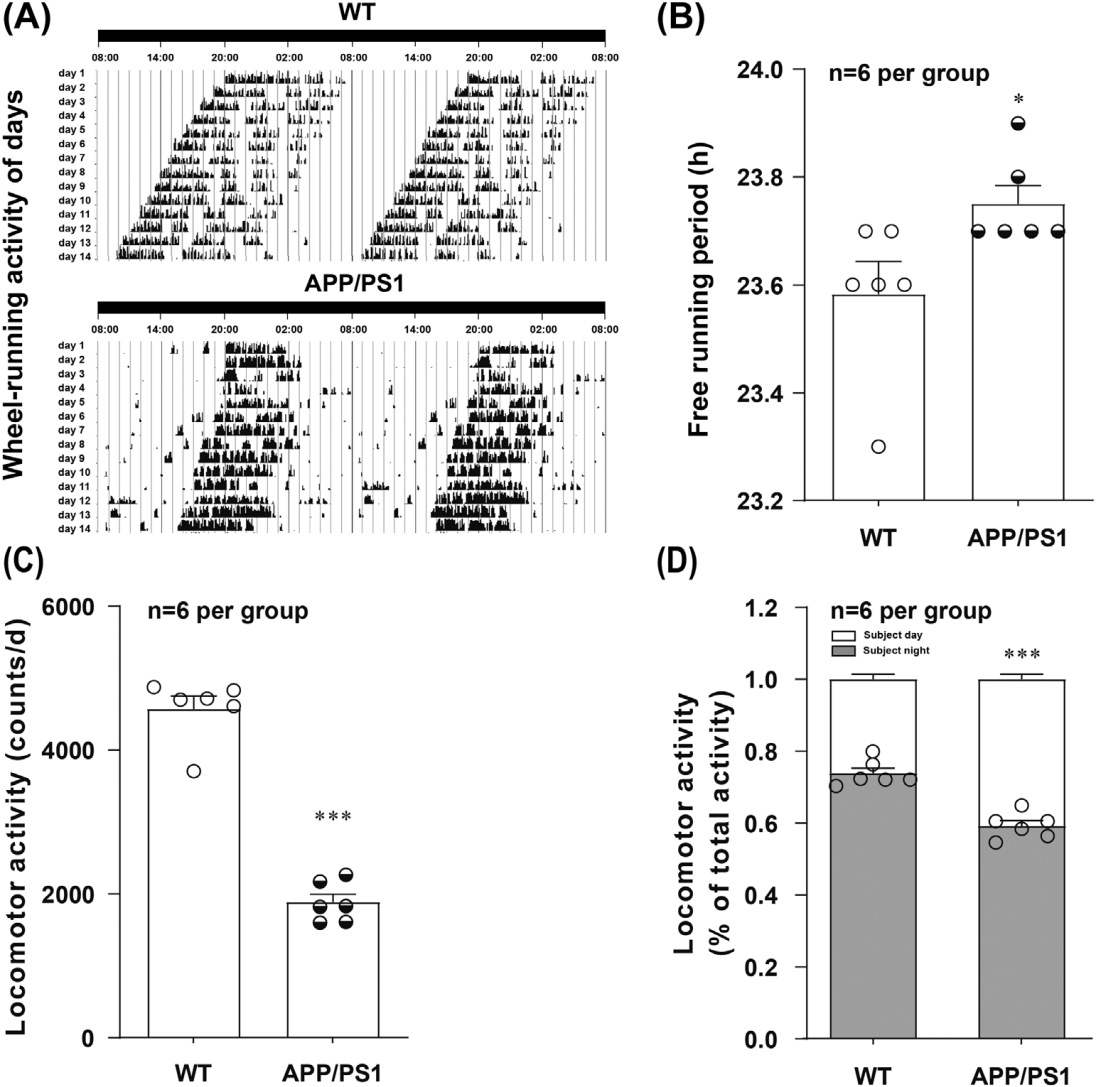
APP/PS1 mice exhibit circadian rhythm disorders. Representative locomotor activity records (A), the free‐running period of the locomotor activity rhythm (*t* = 2.411) (B), locomotor activity (*t* = 12.74) (C), and the ratio of the subjective night/total activity (*F*_(1,20)_ = 99.00) (D) of WT and APP/PS1 groups of mice, respectively (*n* = 6 per group). *p < 0.05, ***p < 0.001 versus corresponding WT group.

We selected 6‐month‐old C57BL/6J and APP/PS1 mice and performed a wheel‐running test to determine whether the circadian rhythm was disrupted in APP/PS1 mice. The results showed that the circadian rhythm of the wheel‐running activity of WT mice mostly occurred at night, and the starting time of daily movement was normal (Figure 1A). However, the wheel‐running activity of the APP/PS1 mice was irregular, as manifested by alterations in the starting time of daily activities (Figure 1A), prolonged free‐running periods (Figure 1B), reduced locomotor activity (Figure 1C) and a decrease in the ratio of the subjective night to total activity (Figure 1D). These results suggest that APP/PS1 mice present disruptions in circadian rhythmicity, which is consistent with previous studies [37, 38].

### 3.2 Rhythmic *Bmal1* mRNA and Protein Levels Are Disrupted in APP/PS1 Mice and Aβ_1‐42_‐Treated HT22 Cells

**FIGURE 2:**
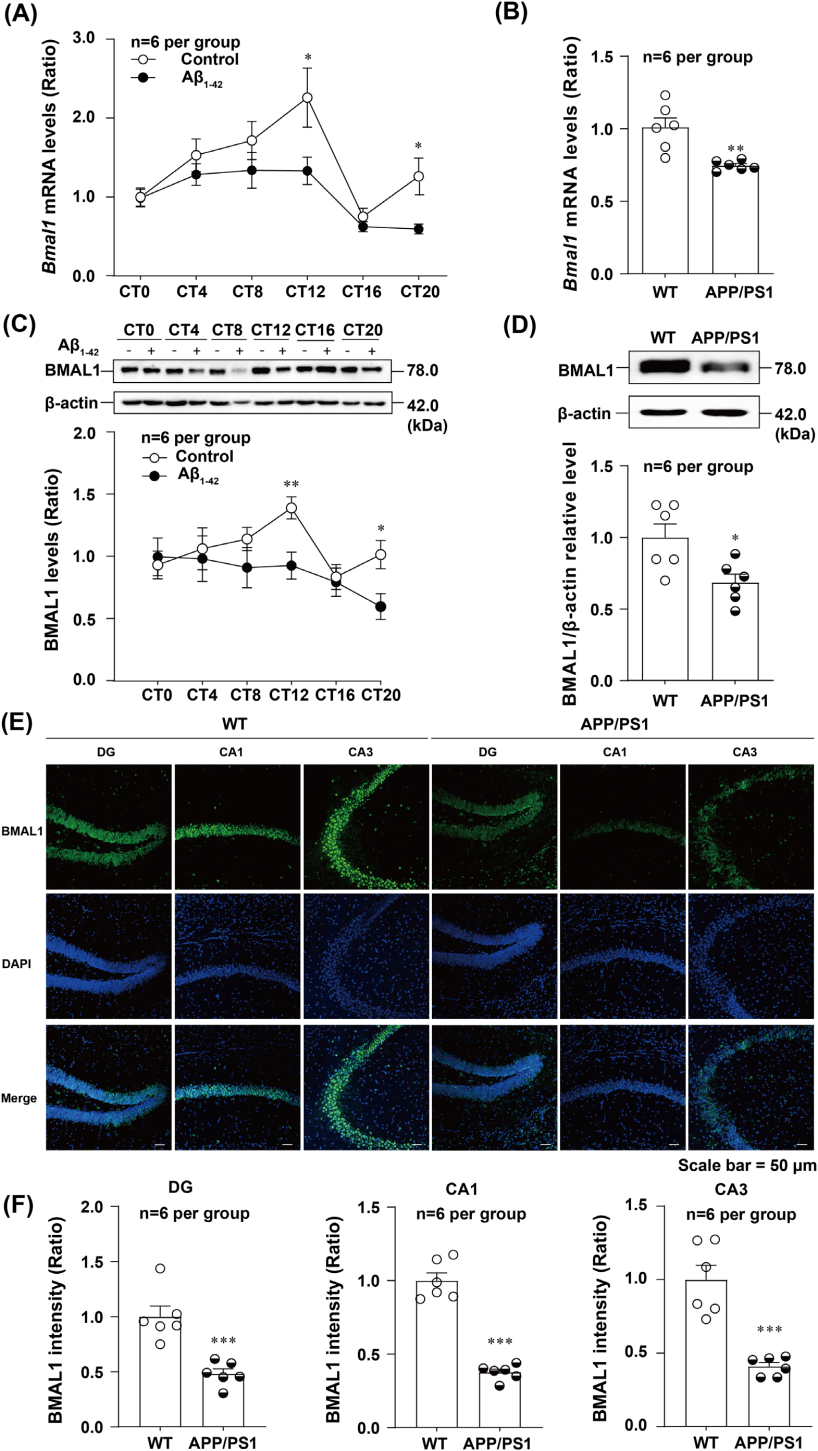
The rhythmicity of *Bmal1* transcript and its protein levels are disrupted in APP/PS1 mice and Aβ_1‐42_‐treated HT22 cells. The statistical results of relative *Bmal1* mRNA (CT12: *t* = 2.238, CT20: *t* = 2.794) (A) and protein (CT12: *t* = 3.282, CT20: *t* = 2.724) (C) levels at the indicated circadian times in Aβ_1‐42_‐treated HT22 cells (*n* = 6 per group). The representative and quantitative analysis of Western blot showed that *Bmal1* mRNA (*t* = 3.988) (B) and protein (*t* = 2.860) (D) levels were significantly decreased at CT12 in APP/PS1 mice (*n* = 6 per group). Representative images (E) and statistical analysis results (F) of immunofluorescent staining showed that BMAL1 intensity was significantly decreased in DG (*t* = 4.916), CA1 (*t* = 10.80) and CA3 (*t* = 5.783) region of APP/PS1 mice (*n* = 6 per group). *p < 0.05, **p < 0.01, ***p < 0.001 versus corresponding Control and WT group.

We explored the effects of Aβ_1‐42_ on *Bmal1* transcript and protein levels in HT22 cells at CT0, CT4, CT8, CT12, CT16 and CT20, using real‐time PCR and Western blot analyses. Consistent with our previous research [11], the results indicated that both *Bmal1* mRNA and protein levels were significantly decreased in Aβ_1‐42_ group at CT12 and CT20 (Figure 2A,C). Furthermore, JTK_CYCLE [34] was used to analyse the rhythmic *Bmal1* mRNA and protein levels. The results showed that *Bmal1* transcript and protein levels exhibited prominent circadian oscillations in the control group, but were disrupted after Aβ_1‐42_ intervention (Table S1A,B). Similarly, our in vivo study revealed that *Bmal1* mRNA, protein levels and fluorescence intensity were significantly decreased at CT12 in the hippocampus of APP/PS1 mice (Figure 2B,D–F). Thus, the circadian rhythmicity of *Bmal1* transcript and protein levels was disrupted both in Aβ_1‐42_‐treated HT22 cells and APP/PS1 mice, as indicated by the decreased levels observed at CT12 and CT20.

### 3.3 DNA Hypermethylation Underlies the Repression of BMAL1 Expression in Aβ_1‐42_‐Treated HT22 Cells

**FIGURE 3:**
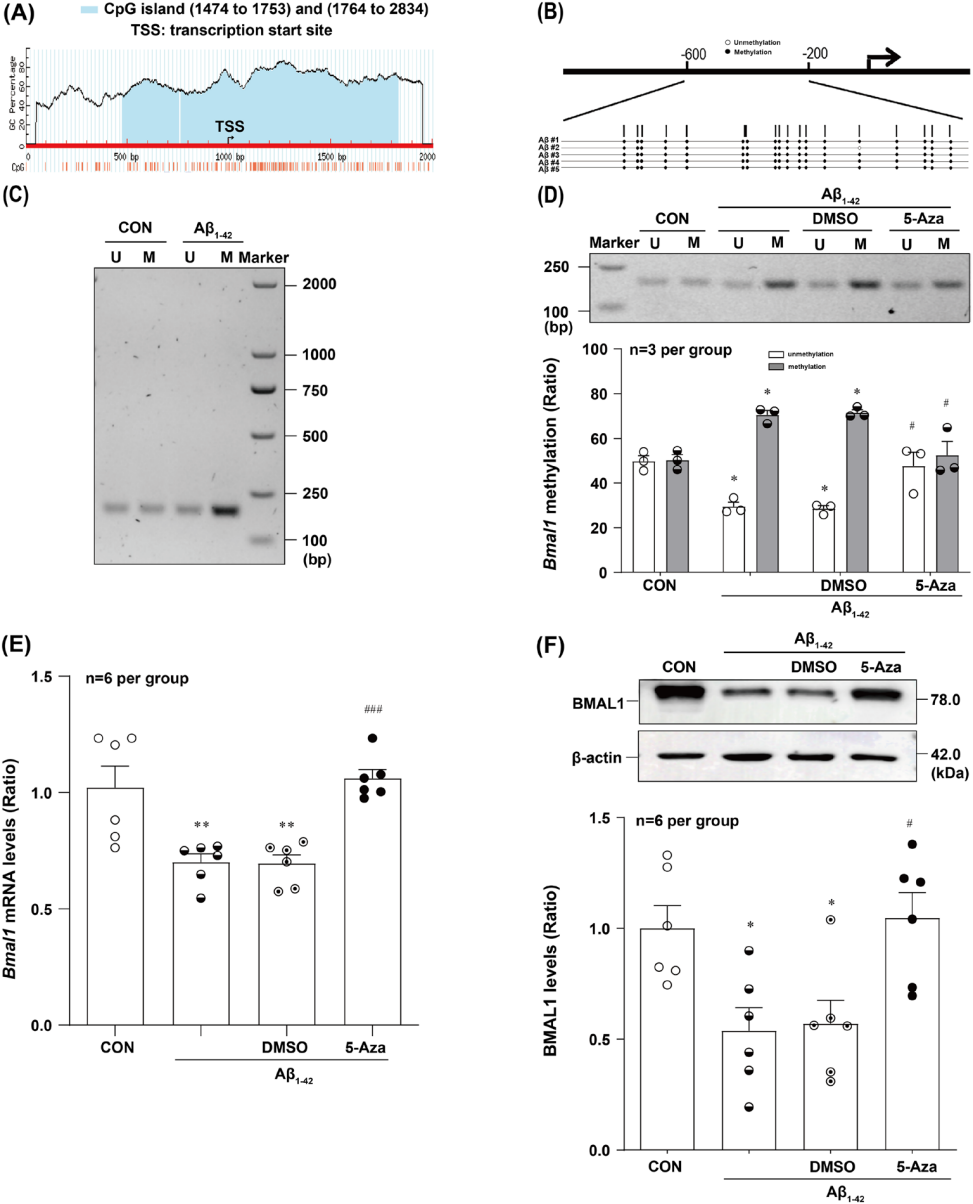
DNA hypermethylation of the promoter of the *Bmal1* gene is essential for reducing its expression in Aβ_1‐42_‐treated HT22 cells. (A) Analysis of the *Bmal1* promotor sequence revealed a noticeable CpG island situated at nucleotides island (1474 to 1753) and (1764 to 2834) (shaded area), based on the algorithm at MethPrimer (www.urogene.org/methprimer). (B) BSP analysis of *Bmal1* 5’‐CpG islands in HT22 cells after Aβ_1‐42_ treatment (*n* = 5 per group). Small rounds, CpG dinucleotides. Methylated (●) and unmethylated (○) CpG dinucleotides were indicated. (C) MSP analysis of *Bmal1* 5′‐CpG island in Aβ_1‐42_‐treated HT22 cells. M, methylated. U, unmethylated. (D) The representative and statistical results of MSP showed that the hypermethylation levels of *Bmal1* was reversed by 5‐Aza intervention in Aβ_1‐42_‐treated HT22 cells (*F*_(3,16)_ = 20.61) (*n* = 3 per group). Pharmacological modulation with 5‐Aza (10 μM) treatment for 24 h restores *Bmal1* transcript (*F*_(3,20)_ = 12.56) (E) and protein level (*F*_(3,20)_ = 6.482) (F) in Aβ_1‐42_‐treated HT22 cells (*n* = 6 per group). *p < 0.05, **p < 0.01 versus corresponding Control group; ^#^ *p* < 0.05, ^###^ *p* < 0.001 versus corresponding vehicle group.

To determine whether Aβ_1‐42_ treatment repressed *Bmal1* transcription via DNA hypermethylation, we performed BSP and MSP to observe *Bmal1* methylation levels in Aβ_1‐42_ exposed HT22 cells. As expected, Aβ_1‐42_ could increase *Bmal1* promoter methylation in HT22 cells (Figure 3A–C). We further examined whether promoter methylation was associated with BMAL1 protein expression by demethylating DNA in HT22 cells using 5‐Aza and determined *Bmal1* transcript and protein levels via real‐time PCR and Western blotting. Treatment of HT22 cells with 5‐Aza reversed the hypermethylation of *Bmal1* (Figure 3D) and subsequently rescued *Bmal1* mRNA and protein levels (Figure 3E,F). Taken together, these data illustrated that Aβ_1‐42_ treatment could lead to *Bmal1* transcriptional repression by methylating its promoter.

### 3.4 The Inhibition of *Bmal1* DNA Methylation Ameliorates Circadian Rhythm Disorders in APP/PS1 Mice

**FIGURE 4:**
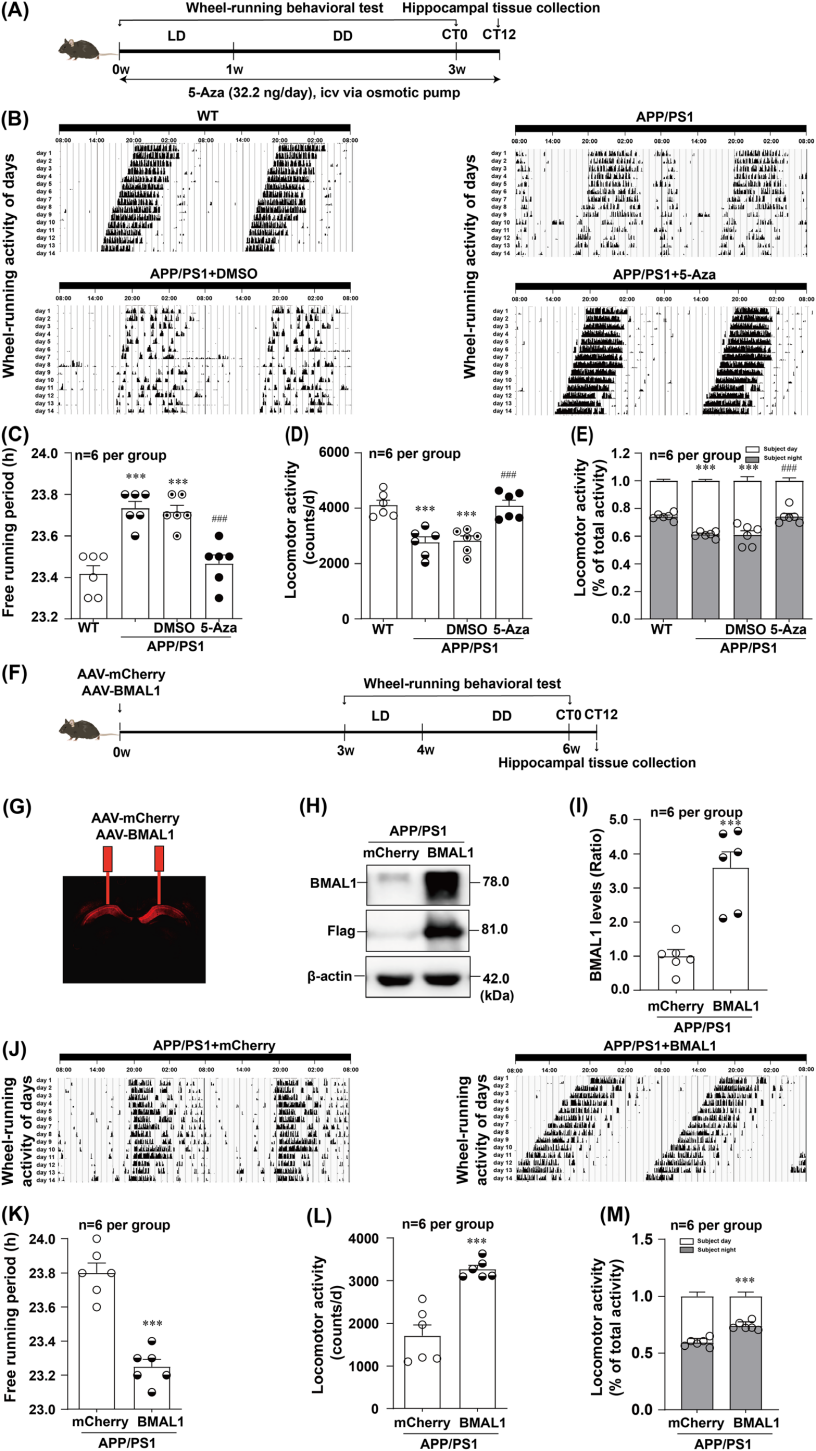
Effects of 5‐Aza on circadian rhythmicity of APP/PS1 mice. (A) Illustration of the experimental timelines showed the 5‐Aza administration and experiments performed. The representative locomotor activity records (B) and the statistical analysis results of the free‐running period of the locomotor activity rhythm (*F*_(3,21)_ = 20.00) (C), locomotor activity (*F*_(3,20)_ = 15.91) (D), and the ratio of the subjective night/total activity (*F*_(3,40)_ = 29.14) (E) showed that 5‐Aza could significantly ameliorated circadian rhythm disorders in APP/PS1 mice (*n* = 6 per group). (F) Illustration of the experimental timelines showed the 5‐Aza administration and experiments performed. Fluorescent image indicated efficient expression of the AAV‐BMAL1 vector in the hippocampus (G). Representative (H) and statistical analysis (I) results of Western blot showed that AAV‐BMAL1 vector increased the BMAL1 levels in the hippocampus of APP/PS1 mice (*t* = 5.149) (*n* = 6 per group). The representative locomotor activity records (J) and the statistical analysis results of the free‐running period of the locomotor activity rhythm (*t* = 7.652) (K), locomotor activity (*t* = 5.749) (L), and the ratio of the subjective night/total activity (*t* = 274.5) (M) demonstrated that AAV‐BMAL1 could significantly alleviated circadian rhythm disruptions (*n* = 6 per group). ***p < 0.001 versus corresponding WT group or APP/PS1 + mCherry group; ^###^ *p* < 0.001 versus corresponding DMSO group.

To explore the effects of 5‐Aza on circadian rhythmicity of APP/PS1 mice, we conducted a wheel‐running behavioural test with 5‐Aza icv injected into the left lateral ventricle of APP/PS1 mice using osmotic micropumps for 21 days (Figure 4A). The results of the sleep–wake cycle (Figure 4B), free‐running period (Figure 4C), locomotor activity (Figure 4D) and the ratio of subjective night activity/total activity (Figure 4E) revealed significant recovery in the APP/PS1 + 5‐Aza group compared with the APP/PS1 + PBS group. These results indicated that 5‐Aza treatment improved circadian disruptions in APP/PS1 mice.

To further determine the effect of BMAL1 on the circadian rhythmicity of APP/PS1 mice, the BMAL1 overexpression vector and mCherry control vector were injected into the hippocampus of APP/PS1 mice 3 weeks before the wheel‐running behavioural test (Figure 4F). The representative fluorescence image of the virus showed its specific expression in the hippocampus (Figure 4G). Western blot analysis revealed that BMAL1 was obviously upregulated in AAV‐BMAL1 mice (Figure 4H,I). Compared with those of APP/PS1 + AAV‐mCherry mice, the results of sleep–wake cycle (Figure 4J), free‐running period (Figure 4K), locomotor activity (Figure 4L) and the ratio of subjective night activity/total activity (Figure 4M) of APP/PS1 + AAV‐BMAL1 mice significantly recovered. These results suggested that BMAL1 overexpression alleviated circadian disruptions in APP/PS1 mice. Taken together, these findings indicate that the inhibition of DNA methylation of *Bmal1* could ameliorate the disturbance of circadian rhythmicity in APP/PS1 mice.

### 3.5 5‐Aza Rescues BMAL1 Expression by Rectifying Its DNA Hypermethylation Through the Inhibition of DNMT1 in APP/PS1 Mice

**FIGURE 5:**
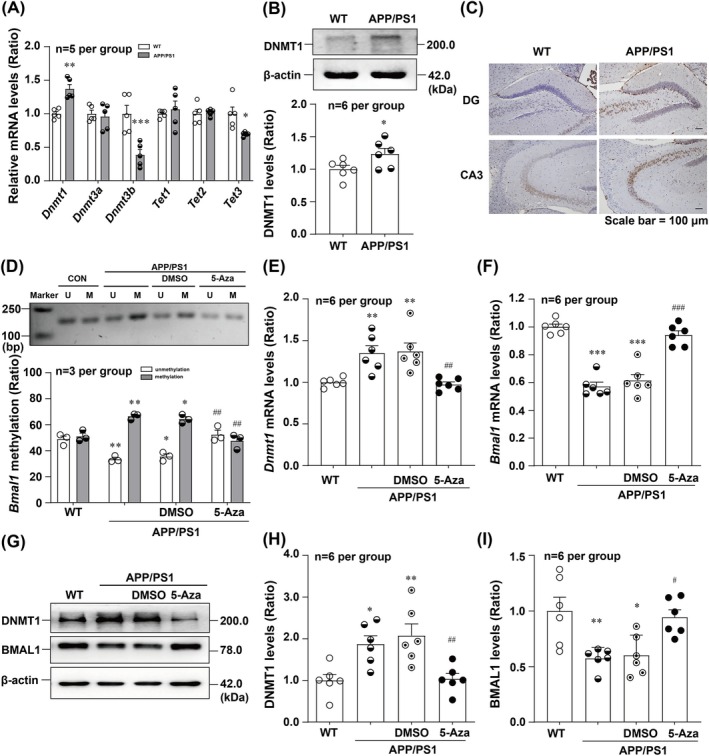
5‐Aza recovers BMAL1 level through DNMT1 inhibition in APP/PS1 mice. (A) Comparison of relative mRNA levels of DNA (de)methylases between WT and APP/PS1 mice at CT12 (*F*_(5,48)_ = 9.953) (*n* = 5 per group). The typical and quantitative analysis results of Western blot demonstrated that DNMT1 (B) protein levels were significantly increased in APP/PS1 mice at CT12 (*t* = 2.298) (*n* = 6 per group). Representative images of immunohistochemical staining for DNMT1 (C) in the hippocampus of two groups of mice at CT12. (D) The representative and statistical results of MSP showed that the hypermethylation levels of *Bmal1* was reversed by 5‐Aza intervention in APP/PS1 mice (*F*_(3,16)_ = 30.81) (*n* = 3 per group). *Dnmt1* transcript (*F*_(3,20)_ = 9.608) (E) and protein levels (*F*_(3,20)_ = 7.902) (G, H) were significantly decreased in 5‐Aza‐treated APP/PS1 mice (*n* = 6 per group). Pharmacological modulation with 5‐Aza treatment for 24 h restored *Bmal1* transcript (*F*_(3,20)_ = 46.62) (F) and protein levels (*F*_(3,20)_ = 7.360) (G, I) in APP/PS1 mice (*n* = 6 per group). *p < 0.05, **p < 0.01, ***p < 0.001 versus corresponding WT group; ^#^ *p* < 0.05, ^##^ *p* < 0.01, ^###^ *p* < 0.001 versus corresponding DMSO group.

DNA methylation is controlled by DNMTs and 10–11 translocation (TET) methylcytosine dioxygenases. DNMTs transfer unmodified cytosine to 5‐methylcytosine (mC), whereas TETs transfer the oxidised 5mC back into 5‐hydroxymethylcytosine and thereby catalyse demethylation [39]. Therefore, we examined the mRNA levels of *Dnmts* and *Tets* and detected higher levels of *Dnmt1*, whereas *Tet3* was expressed at lower levels in APP/PS1 mice than in the WT group (Figure 5A). Furthermore, our Western blot results showed that the expression level of DNMT1 was significantly increased in the hippocampus of APP/PS1 mice (Figure 5B). Similarly, the immunohistochemical staining results revealed that a significantly greater number of DNMT1‐positive cells were present in the hippocampus of APP/PS1 mice than in those of WT mice (Figure 5C). We explored whether 5‐Aza restored BMAL1 expression via the inhibition of DNMT1 by examining *Dnmt1 and Bmal1* mRNA and protein levels. First, the representative and statistical results of MSP indicated that 5‐Aza could reverse the hypermethylation of *Bmal1* in APP/PS1 mice (Figure 5D). Importantly, 5‐Aza reversed the significantly higher level of *Dnmt1* transcript to that observed in WT mice (Figure 5E) and downregulated DNMT1 protein expression (Figure 5G,H). Consistent with our in vitro results, *Bmal1* mRNA (Figure 5F) and protein levels (Figure 5G,I) were restored upon 5‐Aza treatment in APP/PS1 mice. Taken together, these results suggest that DNMT1 is involved in BMAL1 downregulation in APP/PS1 mice.

### 3.6 rmIL‐17A Increases DNMT1 Expression via MAPK Pathway in Aβ‐Treated HT22 Cells

**FIGURE 6:**
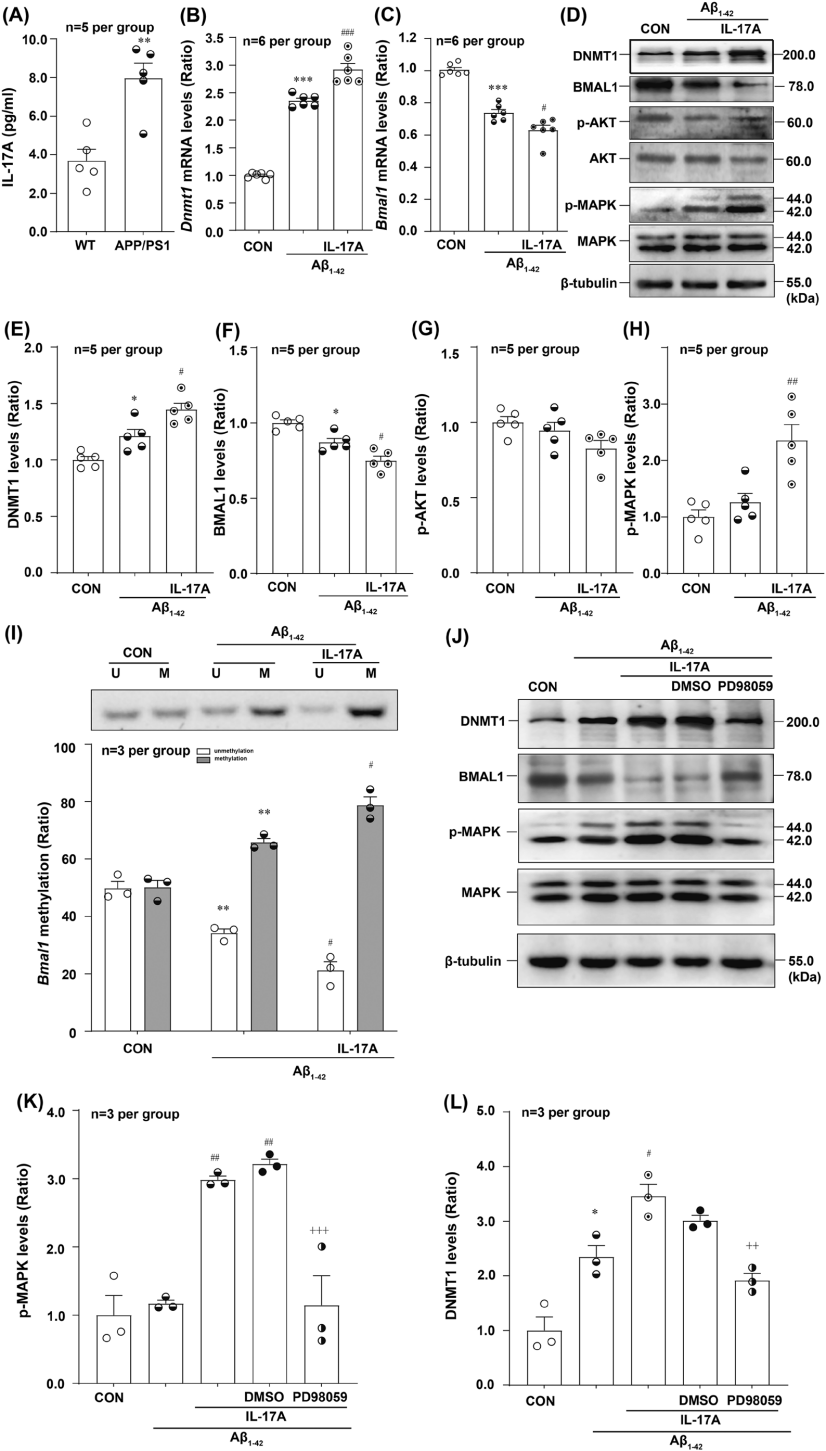
rmIL‐17A contributes to DNMT1 upregulation through MAPK pathway in Aβ‐treated HT22 cells. (A) The statistical results of ELISA indicated that the CSF level of IL‐17A was significantly increased in APP/PS1 mice (*t* = 4.337) (*n* = 5 per group). rmIL‐17A could significantly promote the decrease of *Bmal1* mRNA (C) (*F*_(2,15)_ = 69.13) (*n* = 6 per group) and its protein (D, F) levels (*F*_(2,12)_ = 23.85) (*n* = 5 per group). PCR and WB analysis results showed that the addition of rmIL‐17A could increase the *Dnmt1* transcript (B) (*F*_(2,15)_ = 203.9) (*n* = 6 per group) and protein (*F*_(2,12)_ = 20.01) (D, E) levels (*n* = 5 per group). (I) The representative and statistical results of MSP indicated that rmIL‐17A induced excessive methylation levels of *Bmal1* in Aβ‐treated HT22 cells (*F*_(2,12)_ = 73.43) (*n* = 3 per group). (D, G and H) Western blotting analysis of the phosphorylation levels of AKT and MAPK (*F*_(2,12)_ = 13.39) in HT22 cells upon Aβ treatment (*n* = 5 per group). (J, K) The representative and statistical results of WB confirmed that P‐MAPK was significantly inhibited by MAPK pathway inhibitor PD98059 (10 μM) (*F*_(4,10)_ = 21.28) (*n* = 3 per group). PD98059 downregulated DNMT1 (*F*_(4,10)_ = 25.56) (J, L) leading to the restoration of BMAL1 levels (J) (*n* = 3 per group) *p < 0.05, ***p < 0.001 versus corresponding Control group; **p < 0.01 versus corresponding Control or WT group; ^#^ *p* < 0.05, ^##^ *p* < 0.01, ^###^ *p* < 0.001 versus corresponding Aβ_1‐42_ group; ^++^ *p* < 0.01, ^+++^ *p* < 0.001 versus corresponding Aβ_1‐42_ + IL‐17A + DMSO group.

Previous study reported that the proinflammatory cytokine IL‐6 increased and activated DNMT1‐dependent methylation of the forkhead box‐containing protein class O3a (FOXO3a) [40]. The inflammatory cytokine IL‐17A is known to induce IL‐6 expression [41], and accumulating evidence suggests a pathological role for IL‐17A in AD. Thus, we speculate that IL‐17A might be responsible for DNMT1 elevation in the AD mouse model. First, our ELISA results revealed that there was a significant increase in IL‐17A levels in the CSF of APP/PS1 mice (Figure 6A). We next evaluated the effect of IL‐17A on the expression of BMAL1. As shown in Figure 6C,D,F, rmIL‐17A significantly decreased *Bmal1* mRNA and protein levels in Aβ_1‐42_‐treated HT22 cells. In contrast, the *Dnmt1* transcript and protein levels were significantly increased in HT22 cells exposed to Aβ_1‐42_ + rmIL‐17A (Figure 6B,D,E). Moreover, we observed increased *Bmal1* promoter methylation in Aβ_1‐42_‐treated HT22 cells upon rmIL‐17A intervention (Figure 6I). These results suggested that rmIL‐17A could increase the downregulation of *Bmal1* transcript and protein levels through excessive methylation.

The effects of rmIL‐17A on two key kinases responsible for DNMT1 expression, phosphorylated (P‐) AKT [42, 43] and P‐MAPK [44, 45, 46] were determined to explore the underlying mechanism of IL‐17A‐induced DNMT1 upregulation. The Western blot results for P‐AKT and P‐MAPK revealed no significant differences between the control and Aβ_1‐42_ group, and only the P‐MAPK level was significantly increased upon rmIL‐17A treatment (Figure 6D,G,H). Thus, we speculate that only the MAPK pathway may participate in IL‐17A‐induced DNMT1 upregulation. Consistently, pharmacological inhibition of MAPK (Figure 6J,K) downregulated DNMT1 (Figure 6J,L) levels.

### 3.7 Neutralisation of IL‐17A Alleviates Circadian Rhythm Disruptions and Increases Protein Levels of BMAL1 in APP/PS1 Mice

**FIGURE 7:**
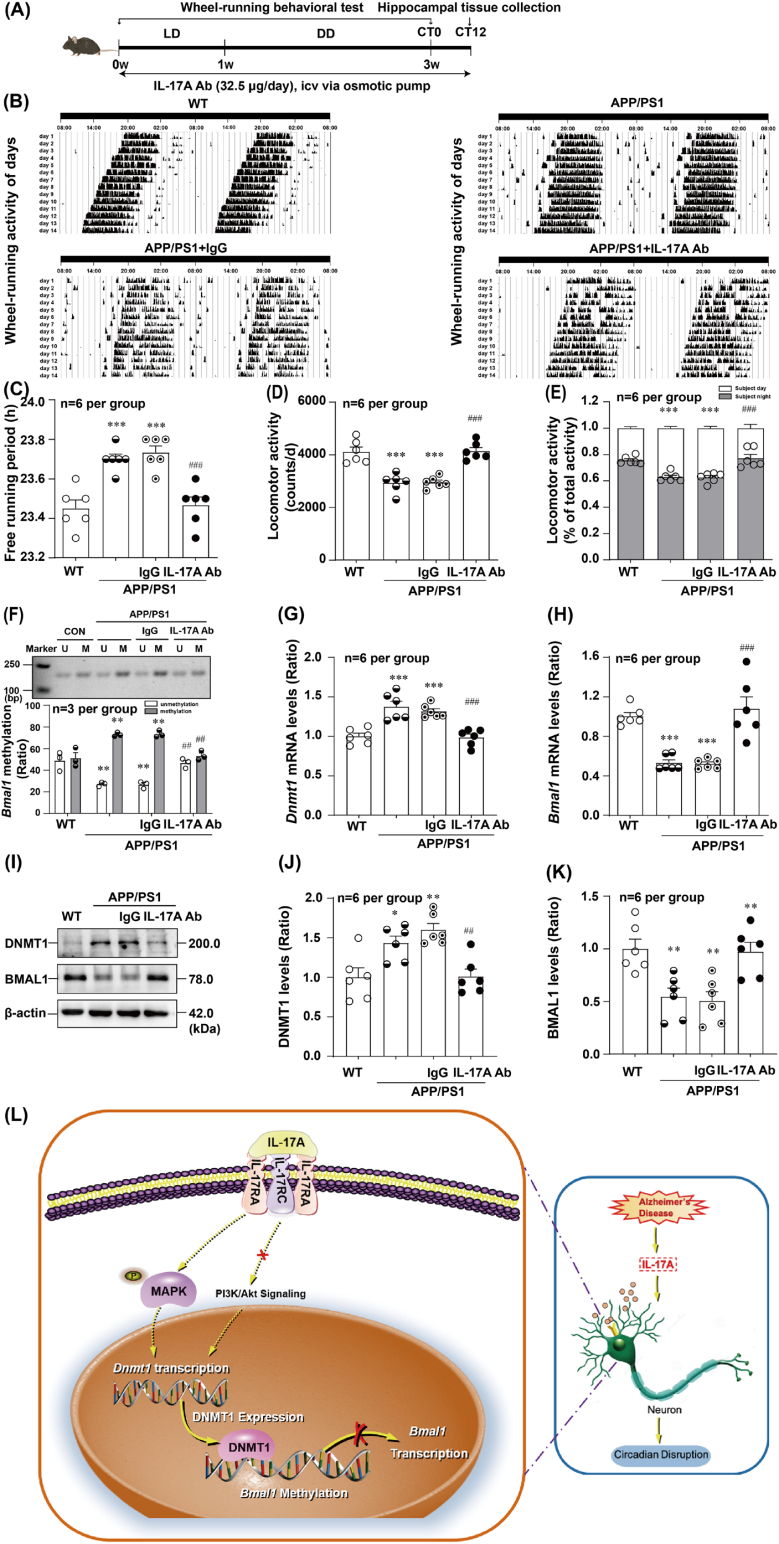
IL‐17A neutralisation ameliorates disturbance of circadian rhythmicity through downregulation the *Bmal1* methylation levels in APP/PS1 mice. (A) Illustration of the experimental timelines showed the IL‐17A Ab administration and experiments performed. The representative locomotor activity records (B) and the statistical analysis results of the free‐running period of the locomotor activity rhythm (*F*_(3,20)_ = 16.68) (C), locomotor activity (*F*_(3,20)_ = 23.50) (D), and the ratio of the subjective night/total activity (*F*_(3,40)_ = 34.73) (E) showed that IL‐17A neutralisation could significantly alleviated circadian rhythm disorders in APP/PS1 mice (*n* = 6 per group). (F) The representative and statistical results of MSP indicated that the hypermethylation levels of *Bmal1* was reversed after IL‐17A neutralisation in APP/PS1 mice (*F*_(3,16)_ = 31.59) (*n* = 3 per group). PCR and WB analysis results showed that IL‐17A neutralisation could reverse the *Dnmt1* transcript (*F*_(3,20)_ = 17.98) (G) and protein (*F*_(3,20)_ = 9.971) (I, J) levels (*n* = 6 per group). IL‐17A neutralisation restored *Bmal1* transcript (*F*_(3,20)_ = 20.42) (H) and protein levels (*F*_(3,20)_ = 9.079) (I, K) in APP/PS1 mice (*n* = 6 per group). (L) Schematic diagram of IL‐17A induces circadian disruptions via epigenetic repression of BMAL1 in APP/PS1 mice. *p < 0.05, **p < 0.01, ***p < 0.001 versus corresponding WT group; ^##^ *p* < 0.01, ^###^ *p* < 0.001 versus corresponding IgG isotype group.

Next, we assessed whether the increased levels of IL‐17A in the CSF of APP/PS1 mice had a detrimental effect on wheel‐running behavioural test performance. For this purpose, the IL‐17A neutralising mAb or isotype control IgG was chronically diffused through osmotic micropumps into the left lateral ventricle of the mice for 21 days (Figure 7A). Strikingly, the results of the sleep–wake cycle (Figure 7B), free‐running period (Figure 7C), locomotor activity (Figure 7D) and the ratio of the subjective night activity/total activity (Figure 7E) showed significant improvements in the APP/PS1 + anti‐IL‐17A group compared with the APP/PS1 + IgG group. These data indicated that anti‐IL‐17A treatment alleviated circadian disturbances in APP/PS1 mice.

The levels of *Bmal1* methylation were significantly decreased in APP/PS1 mice after IL‐17A neutralisation (Figure 7F). Furthermore, the effects of IL‐17A neutralisation on *Dnmt1* and *Bmal1* transcript and protein levels were determined. As shown in Figure 7G,I,J, IL‐17A neutralisation significantly decreased *Dnmt1* mRNA and protein levels. *Bmal1* mRNA and protein levels were significantly increased in the APP/PS1 + anti‐IL‐17A group compared with the APP/PS1 + IgG group (Figure 7H,I,K). Taken together, these results demonstrated that IL‐17A neutralisation ameliorated circadian rhythm disorders through *Bmal1* hypomethylation in APP/PS1 mice.

## 4 Discussion

Circadian disruptions and neuroinflammation are highly prevalent among people with AD, and accumulating evidence suggests that IL‐17A exacerbates the process of AD by promoting Aβ accumulation, Tau hyperphosphorylation, neuronal loss and APP amyloid pathway [25, 47]. Nevertheless, the impact of IL‐17A on circadian disruption‐induced AD remains elusive.

In this study, we showed that elevated levels of IL‐17A downregulated BMAL1 via excessive methylation, leading to circadian disruptions in APP/PS1 mice. We revealed that the excessive methylation of the *Bmal1* promoter through the MAPK pathway and identified the upregulation of DNMT1 as the underlying epigenetic repressive mechanism. Therefore, our findings highlight, for the first time, a mechanism linking IL‐17A to altered expression of BMAL1, which contributes to circadian disturbances in the AD mouse model.

Circadian rhythm dysfunction is a fundamental factor contributing to exacerbating AD, and understanding its mechanism could help in the identification of therapeutic targets for this disease. In vivo research has indicated that AD mice present disrupted cage activity and circadian regulation [11, 37, 38, 48]. Consistent with these findings, we observed that 6‐month‐old APP/PS1 mice presented circadian rhythm disorders. The hippocampus is central to AD pathology and memory, and the molecular clock in which is crucial for day‐night differences in learning and memory [49, 50]; therefore, we focused on the direct impact of BMAL1 expression in the hippocampus on AD‐induced circadian rhythm disorders. Our study provides evidence that the hippocampus is a centrally situated circadian oscillator that is sensitive to AD‐induced circadian disruptions.

The circadian clock is composed of a transcriptional‐translational feedback loop that regulates daily physiology. The circadian locomotor output cycles kaput (CLOCK)‐BMAL1 heterodimer initiates the transcription of *Per* and *Cry*. The PER‐CRY heterodimer negatively modulates the transcription of their cognate genes [51]. Our previous studies showed that Aβ_31‐35_ disrupted the circadian rhythms of *Per1* and *Per2* mRNA and protein levels both in the suprachiasmatic nucleus (SCN) [52] and hippocampus [11, 52] of C57BL/6 mice. In addition, *Clock* mRNA and protein levels are higher in APP/PS1 mice than in WT mice [53]. Among the circadian clock genes described above, *Bmal1* is the only irreplaceable clock gene that modulates all modulating whole rhythmic behaviours [54]; thus, we focused on the role of BMAL1 in the development of the AD mouse model and its molecular mechanisms. *Bmal1*, a core positive clock gene, reportedly plays a fundamental role in regulating circadian rhythm. The deletion of *Bmal1* contributes to the complete abolition of both behavioural and molecular circadian rhythms in mice [54]. Furthermore, *Bmal1* deletion in mice could lead to memory deficits [55]. On the other hand, Aβ could induce BMAL1 degradation, resulting in circadian rhythm disruptions [9, 12]. Moreover, alteration in *Bmal1* transcription was observed in the brains of AD patients when compared with those in the control group [56]. Consistent with these observations, we also detected that the circadian rhythmicity of *Bmal1* transcript and protein levels was disrupted in both Aβ‐treated HT22 cells and APP/PS1 mice, as indicated by the significant decreases observed at CT12 and CT20. Studies have shown that DNA methylation of the *Bmal1* gene could lead to its transcriptional silencing, which is critical for interfering with circadian rhythms [15]. DNA hypermethylation of the *Bmal1* gene in superior frontal gyrus tissues of AD patients was observed and was positively correlated with changes in cognitive measures and night waking [14]. However, the underlying mechanisms of *Bmal1* hypermethylation in the AD model still remain unclear. Our study also revealed that the *Bmal1* promoter was hypermethylated in both Aβ‐treated HT22 cells and APP/PS1 mice, and for the first time, we provided ample evidence for the specific molecular mechanisms of *Bmal1* hypermethylation in an AD mouse model. We have confirmed that 5‐Aza rescues BMAL1 expression through reversing its hypermethylation and ameliorates circadian rhythm disorders in APP/PS1 mice. However, it is not clear whether the improvement of circadian rhythmicity in the APP/PS1 mouse model by 5‐Aza is through the effect on the *Bmal1* gene. Our results showed that the specific overexpression of BMAL1 in the hippocampus improved circadian disruptions in APP/PS1 mice, demonstrating that 5‐Aza could ameliorate circadian rhythm disruptions in APP/PS1 mice through *Bmal1* demethylation. Consistent with this, a previous study has reported that 5‐Aza demethylated the *Bmal1* promoter and thus suppressed its transcription in primary cultured human aortic endothelial cells [57]. To further determine the mechanisms of DNA hypermethylation of *Bmal1*, we then measured the mRNA levels of DNMTs and TETs, which are responsible for DNA methylation and found that the *Dnmt1* mRNA level in APP/PS1 mice was significantly higher than that in WT mice. Furthermore, 5‐Aza treatment restored BMAL1 levels through the inhibition of DNMT1, resulting in the amelioration of circadian rhythm disorders in APP/PS1 mice. DNMT1 inhibition is due to the formation of a covalent complex between 5‐Aza and DNMT1, resulting in DNMT1 inactivation [58]. These data showed that DNMT1 could negatively regulate the circadian rhythm of AD model mice. Increased transcript and protein levels of DNMT1 were observed both in AD patients [59] and APP/PS1 mice [18] and in a high‐methionine diet‐induced AD model [17]. However, a small number of studies revealed a decreased level of *Dnmt1* or even no difference in its mRNA level in different AD models [60, 61]. This variation may be attributed to the diversity of AD models.

IL‐17A exacerbates neuroinflammation by triggering lymphocyte infiltration, neutrophil migration through the blood–brain barrier, and the secretion of other inflammatory factors such as IL‐6, TNF‐α and IL‐1β [25, 41, 47], which further promote AD‐like pathology and cognitive deficits in an AD mouse model [62]. Several studies have demonstrated that cytokine secretion is under circadian control in various disease modes [63, 64, 65], but the effects of the released cytokines on the circadian rhythm are not yet clear. Studies have shown that the IL‐6/JAK2/STAT3 pathway induces elevated expression of DNMT1 in both lung cancer stem cells [66] and neural stem cells [18]. However, no studies have discussed the relationship between DNMT1 and IL‐17A in AD. Thus, we speculate that IL‐17A might be responsible for DNMT1 upregulation in AD mice. Studies have suggested that the MAPK pathway is responsible for *Dnmt1* transcription [53] and that it could be activated by IL‐17A [67, 68, 69]. In addition, earlier evidence indicated that AKT could stabilise DNMT1 and that the IL‐8/AKT pathway stabilised DNMT1 protein expression [42, 70]. Thus, the effects of rmIL‐17A on these two key kinases, which are responsible for DNMT1 expression, were examined. We observed that only MAPK activation by IL‐17A upregulated DNMT1 in Aβ‐treated HT22 cells, which was consistent with previous results. Therefore, we propose that *Bmal1* epigenetic silencing is due to the IL‐17A‐MAPK signalling pathway‐induced methylation via elevated DNMT1 expression. Our study further showed that IL‐17A neutralisation could reverse BMAL1 downregulation via DNA methylation and alleviate circadian rhythm disorders. These data indicate that IL‐17A negatively modulates the circadian rhythm of AD model mice. Furthermore, MAPK pathway activation‐triggered DNMT1 expression provides new insights into the role of IL‐17A in maintaining the circadian rhythmicity of AD mice.

## 5 Conclusions

In summary, our study shows that the IL‐17A‐MAPK pathway regulates the circadian rhythm by altering DNMT1 in Aβ‐treated HT22 cells and that neutralisation of IL‐17A increases the transcription of *Bmal1* via downregulation of DNA methylation, thus ameliorating circadian rhythm disruption in APP/PS1 mice. These data indicate that the IL‐17A‐MAPK pathway plays a crucial role in regulating *Bmal1* methylation by activating DNMT1, resulting in circadian rhythm disruptions in APP/PS1 mice, which provides a basis for speculating on potential mechanisms and therapeutic strategies for AD.

## Author Contributions

**Ting Liu:** investigation, methodology, funding acquisition, visualization, formal analysis, writing – original draft. **Tian Mao:** investigation, methodology, project administration, validation. **Jinxuan Fan:** investigation, validation, visualization. **Yanjun Shen:** conceptualization, data curation. **Lingxia Xue:** investigation, data curation. **Kaili Du:** project administration, software. **Yang Li:** investigation, validation. **Li Wang:** supervision, Funding acquisition, writing – review and editing. **Xiaohui Wang:** conceptualization, funding acquisition, project administration, Supervision, writing – review and editing.

## Conflicts of Interest

The authors confirm that there are no conflicts of interest.

## Supporting information


Appendix S1

## Data Availability

All the data supporting this study are available in the manuscript. Raw data are available from the corresponding authors upon reasonable request.
