# Supplementary material for: IL‐17A Induces Circadian Disruptions Through the Epigenetic Repression of BMAL1 in Mice With Alzheimer's Disease
Source: J Cell Mol Med. 2025 Apr 10;29(7):e70546. doi: 10.1111/jcmm.70546 (PMC11984323; doi:10.1111/jcmm.70546)
Supplement: Supplementary file 1 — Appendix S1 [file JCMM-29-e70546-s001.docx]

*Supplementary Materials*

**Supplementary tables**

**Supplementary Table 1 JTK_CYCLE results of *Bmal1* mRNA between Control and Aβ_1-42_ treatment.**

| Sample type | Item | Group | JTK_CYCLE  P_value | Circadian  JTK_CYCLE |
| --- | --- | --- | --- | --- |
| in vitro | Bmal1 | Control | 0.001 | Yes |
| in vitro | Bmal1 | Aβ_1-42_ | 0.058 | No |

**Supplementary Table 2 JTK_CYCLE results of BMAL1 protein level between Control and Aβ_1-42_ treatment.**

| Sample type | Item | Group | JTK_CYCLE  P_value | Circadian  JTK_CYCLE |
| --- | --- | --- | --- | --- |
| in vivo | BMAL1 | Control | 0.023 | Yes |
| in vivo | BMAL1 | Aβ_1-42_ | 0.588 | No |

**
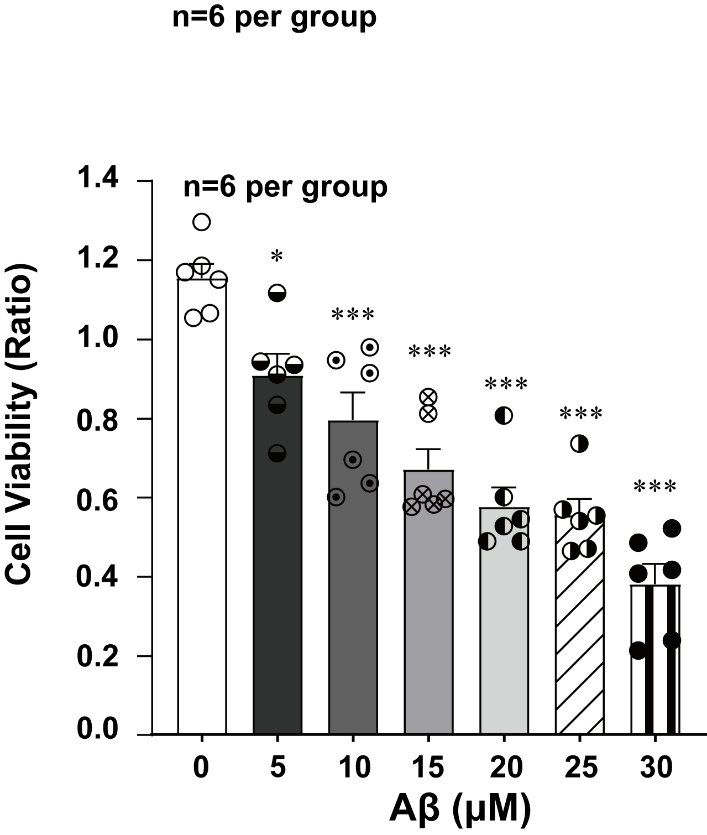
Supplementary figures**

**Supplementary Figure 1. Effect of Aβ_1-42_ on the cell viabilities in HT22 cells.** The results of Cell Counting Kit-8 assay showed that Aβ could aggravate the cell viabilities of the HT22 cell with 24-h treatments (n = 6 per group). Data are presented as mean ± SEM; *p < 0.05, ***p < 0.001 versus with corresponding control group.

**
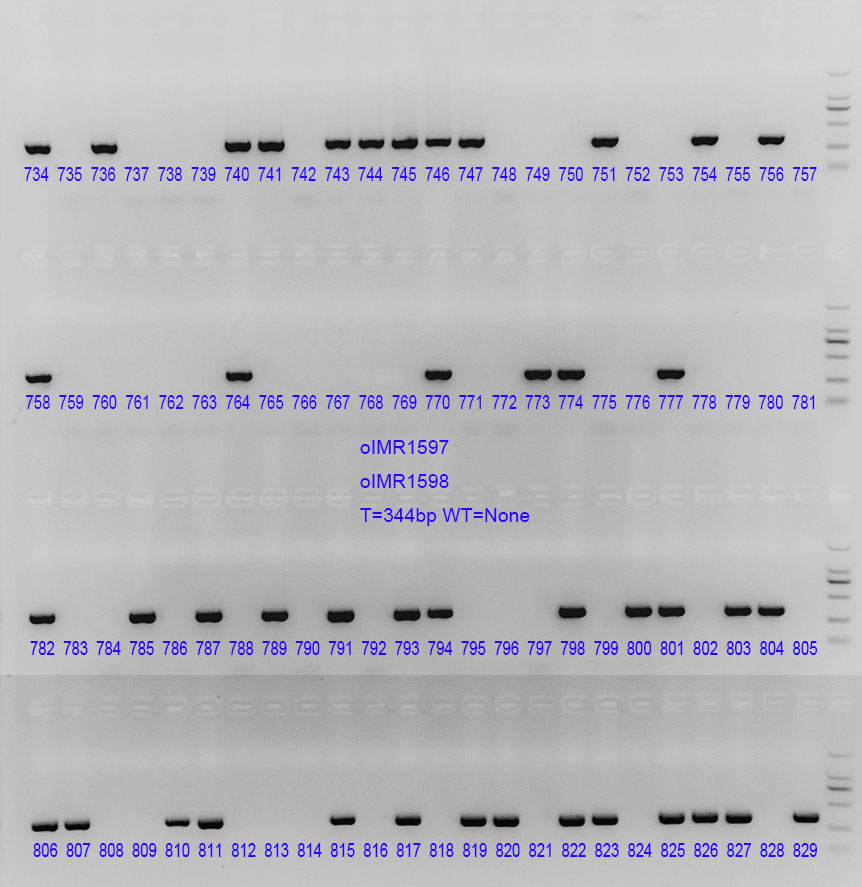
**

**APP 344 bp**

**
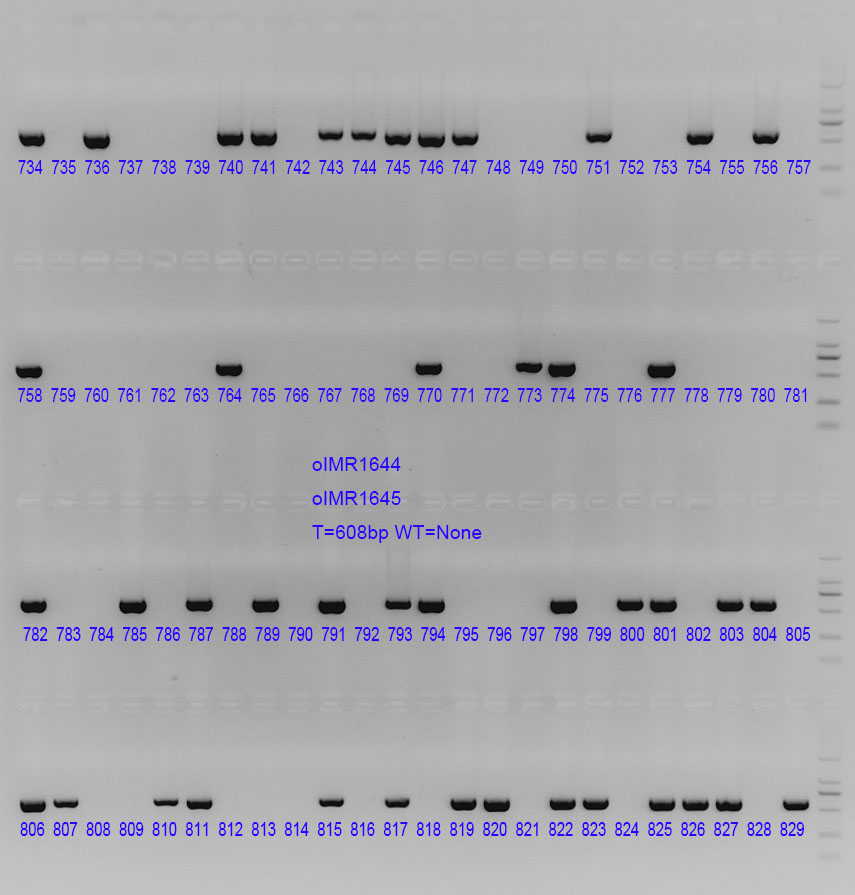
**

**PS1 608 bp**

**
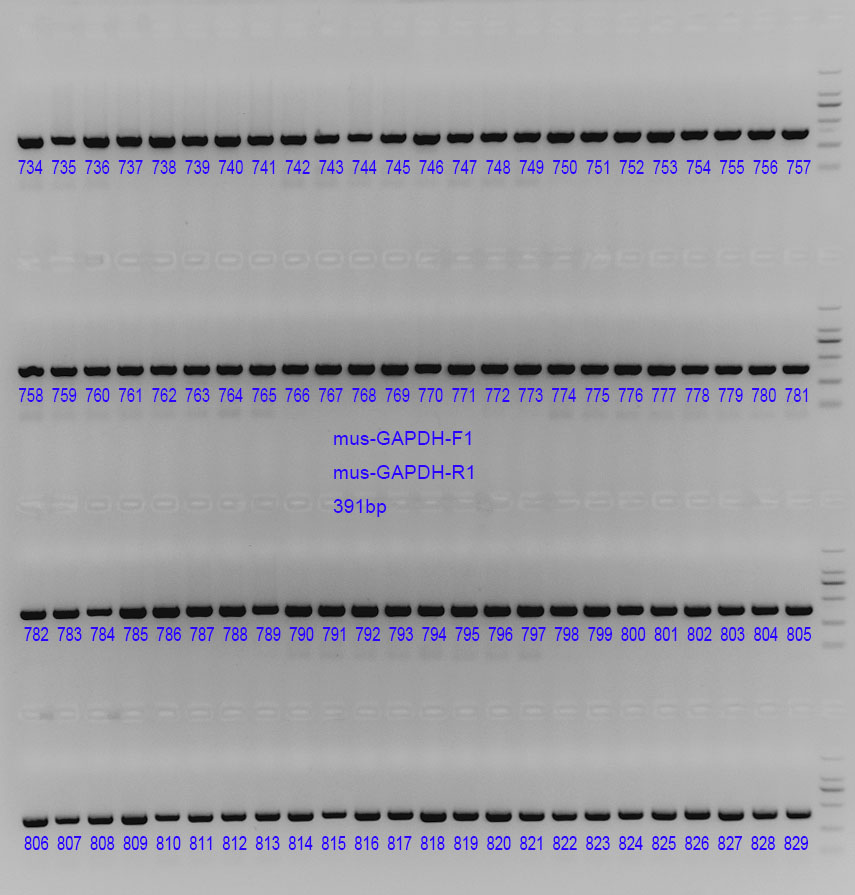
**

**GAPDH 391 bp**

**Supplementary Figure 2. The results of gel electrophoresis in APP/PS1 transgenic mice were identified by PCR.** Mouse tail DNA was extracted from the offspring of the first established mouse, amplified by PCR, and 1% agarose gel electrophoresis.

**
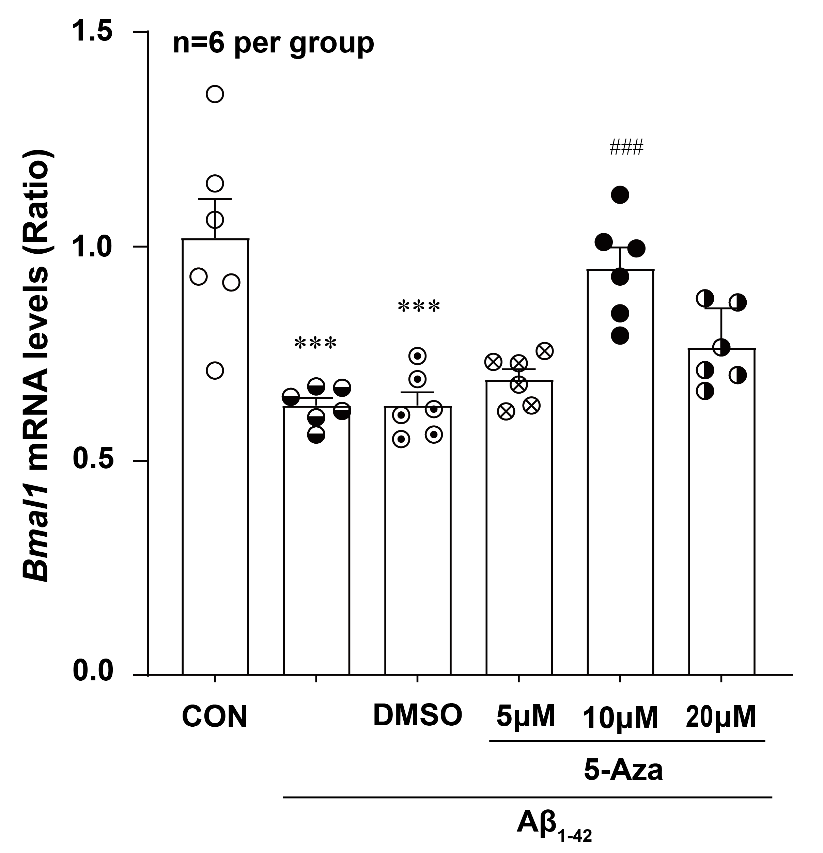
**

**Supplementary Figure 3. Effects of different dosage of 5-Aza on *Bmal1* mRNA levels in Aβ-treated HT22 cells.** mRNA levels of *Bmal1* were only restored upon 10 μM 5-Aza + Aβ_1-42_ group compared with Aβ_1-42_ group in HT22 cells (n = 6 per group). Data are presented as mean ± SEM; ***p < 0.001 versus with control group; ### p < 0.001 versus with DMSO group.
